# Description of third instar larvae of *Ceratitis fasciventris*, *C. anonae*, *C. rosa* (FAR complex) and *C. capitata* (Diptera, Tephritidae)

**DOI:** 10.3897/zookeys.540.10061

**Published:** 2015-11-26

**Authors:** Gary J. Steck, Sunday Ekesi

**Affiliations:** 1 Florida Department of Agriculture and Consumer Services, Division of Plant Industry, 1911 SW 34th Street, Gainesville, FL 32614-7100 USA; 2 International Centre of Insect Physiology and Ecology (ICIPE) PO Box 30772-00100 GPO, Nairobi, Kenya

**Keywords:** Mediterranean fruit fly, immature stages, taxonomy, identification

## Abstract

Third instar larvae of members of the *Ceratitis* FAR complex, including *Ceratitis fasciventris* (Bezzi), *Ceratitis anonae* Graham, and *Ceratitis rosa* Karsch are described and compared with those of *Ceratitis capitata* (Wiedemann). Diagnostic characters, such as presence vs. absence of a secondary tooth on the mandibles, previously used to separate *Ceratitis capitata* from *Ceratitis rosa*, are shown to vary in each species. Significant variation in diagnostic morphological characters among populations of *Ceratitis rosa* from east and south Africa is documented; however, the differences are not simply congruent with the R1 and R2 designations based on other studies. Quantitative measures of numerous morphological characters are consistently smaller in the larvae of *Ceratitis fasciventris* and distinguish them from other species of the FAR complex. Larvae of *Ceratitis capitata* can be distinguished from those of the FAR complex by characters such as absence of accessory plates of the oral ridges, the shape of the anterior spiracle, and the pattern of dorsal spinules. Previous studies indicated that absence of accessory lobes separate the genus *Ceratitis* from *Bactrocera*, but this is shown to be incorrect, as accessory lobes are in fact present in several species of *Ceratitis*.

## Introduction

Members of the *Ceratitis* FAR complex, including *Ceratitis fasciventris* (Bezzi), *Ceratitis anonae* Graham, and *Ceratitis rosa* Karsch, are serious agricultural pests in large parts of Africa. Understanding of the species taxonomy is important to determining host plant relationships, pest management practices, knowledge of geographical distribution, and quarantine and related plant protection issues. In recent years, the taxonomy of species of the *Ceratitis* FAR complex has been clarified by careful study of adult morphology, and on-going morphological, genetic, and physiological studies suggest that additional, previously unrecognized species may be present (De Meyer 2001, De Meyer and Freidberg 2006, Virgilio et al. 2008, Virgilio et al. 2013). The present morphological study of the immature stages is intended to augment and possibly corroborate the differences seen among adult and various other biological parameters. In particular, this study was originally prompted by a problem in identifying larvae that had been intercepted alive infesting fresh peppers (*Capsicum chinense* Jacq. ‘Habanero’) that had been shipped from the Netherlands and intercepted in Miami, Florida in August, 2004. It wasn’t clear whether they were *Ceratitis rosa* or *Ceratitis capitata* (Wiedemann) because of poorly documented overlapping morphological variation in these two species that confounded the identification process. Years later, in a telling of this dilemma during general discussion at the first meeting of the International Atomic Energy Agency’s Coordinated Research Project on fruit fly cryptic species complexes in Vienna in 2010, the senior author learned that there existed research colonies of various *Ceratitis* species at ICIPE from which immature stages could be obtained, and a collaboration to do a morphological study was developed. It was quickly discovered that larvae from the Kenyan colony of *Ceratitis rosa* differed in a significant feature from those of a South African colony as described by Carroll (1998). This prompted a broader study of immature stages of all three species of the FAR complex and their various populations as described here.

## Materials and methods

One goal of this paper is to describe morphological variation among geographic populations of *Ceratitis rosa*. To that end and for ease of comparison, Carroll’s (1998) well-written description of mature 3^rd^ instar larvae is repeated verbatim below (in italics) and any differences observed in the populations in the present study are given in parentheses in bold, normal font. Descriptions of other instars, pupae and eggs of most fruit fly species, including important pests, are generally unavailable for comparison. Full data is given in corresponding tables. Some features detailed by Carroll (1998) were not examined in this study and those portions of her description are not repeated. These include most of the sensillae (on the stomal organ, maxillary palp, cephalic segment, Keilin’s organ, and other body parts), and vestigial spiracular openings. Most of these sensillae are only seen with a scanning electron microscope (SEM), they are often difficult to find, and may be variably expressed. They are generally highly conserved among cyclorrhaphan Diptera and have not been found useful in alpha taxonomy. Carroll did not provide measurement data on the cephalopharygneal skeleton, but these data are provided here because they do show differences among species, and they reliably separate the instars. The descriptions and measurements follow the terminology of Teskey (1981), Steck and Malavasi (1988), Steck and Wharton (1988), and Carroll (1998). New figures are provided for most of the same features illustrated in Carroll (1998).

Corresponding descriptions were made of larvae of other species of the FAR complex, namely *Ceratitis anonae* (minimally described by Silvestri (1914) and included in the key of White and Elson-Harris (1992)) and *Ceratitis fasciventris* (not described previously). A further goal is to provide means of identifying larvae of these species morphologically, if possible. Additionally, equivalent observations were made of *Ceratitis capitata* larvae, as this important pest is likely to be encountered in the same host plants and geographic regions as members of the FAR complex.

### The samples in this study include

*Ceratitis rosa* R1 (“hot”): KENYA, from a colony at the International Centre of Insect Physiology and Ecology (ICIPE) 20 May 2013, originating in coastal region: Mwanjamba, 04°18'21"S; 39°29'88"E, 106 m above sea level; host plant: guava; 5^th^ generation. SOUTH AFRICA: from a colony at Citrus Research International (CRI), originating from Nelspruit, 25°27'08.19"S; 30°58'11.27"E, 11 Nov 2013, host: loquat, *Eriobotrya japonica* (Thunb.) Lindley, collector J-H. Daneel; 9^th^ generation.

*Ceratitis rosa* R2 (“cold”): KENYA: from a colony at ICIPE 3 April 2001 (originating in highlands, unknown number of generations in laboratory), and December 2010, originating in highlands: Kithoka, 00°05'59"N; 037°40'40"E and 1425 m above sea level; host plant: mango; 6^th^ generation; SOUTH AFRICA: from a colony at CRI, originating from Pretoria, 25°45'13.7"S; 28°13'45"E, 25 Feb 2014, host: jambos, *Syzygium jambos* (L.) Alston, collector J-H. Daneel; 6^th^ generation; and from a colony at Stellenbosch University, originating from Stellenbosch, 33°56'10.99"S; 18°51'56.186"E, April 2013, host: kei apples, *Aberia caffra* Hook f. & Harv., collector Pia Addison.

*Ceratitis anonae*: KENYA: from a colony at ICIPE, 4 Feb 2011, originating in Kakamega forest, Western Kenya, 0°16'0"N; 34°52'60"E and altitude of 1603 m above sea level; host plant: *Antiaris toxicara* (Pers.) Lesh (Moraceae); 101^st^ generation.

*Ceratitis fasciventris*: KENYA: from a colony at ICIPE, 7 Feb 2011, originating in Ruiru, Central Kenya, 1°8'31.9"S; 36°57'23.5"E and altitude of 1612 m above sea level; host plant: *Coffea arabica* L. (Rubiaceae); 92^nd^ generation.

*Ceratitis capitata*: KENYA: from colonies at ICIPE, 3 April 2001 (source material unknown) and 4 Feb 2011, originating in Ruiru, Central Kenya, 1°8'31.9"S; 36°57'23.5"E and altitude of 1612 m above sea level; host plant: *Coffea arabica* L. (Rubiaceae); 231^st^ generation; GUATEMALA: from a colony (probably USDA-APHIS), July 1987; USA, Hawaii: from a colony, Steiner laboratory, 1957; USA, Florida: Miami via Netherlands, 2004 ex habanero peppers (*Capsicum chinense* Jacq. ‘Habanero’), Division of Plant Industry accession # E2004-6626; and various samples of dead, cold-treated larvae intercepted in clementines (*Citrus reticulata*) from Spain.

Voucher specimens of all African FAR colonies were verified morphologically based on adult males (De Meyer et al. 2015) and the Kenyan colonies were also genotyped for both sexes (De Meyer, personal communication 31 March 2015).

Larvae were killed in hot water and preserved in 70% ethanol or isopropanol. Specimens intended for SEM examination were sonicated for 30 seconds, then dehydrated in an ethanol series, followed by ethyl acetate, then air-dried, mounted on stubs, sputter-coated with gold-palladium, and examined in a JEOL JSM-5510LV SEM at FDACS/DPI, Gainesville, FL. Measurements derived from stub-mounted specimens were made from SEM photographs and calibrated using the embedded scale bar.

Specimens intended for examination under dissecting and compound optical microscopes were macerated overnight in 10% NaOH at room temperature. Once cleared, they were temporarily slide mounted in glycerin and positioned to allow measurements as needed. Most measurements were made manually using an eyepiece reticle calibrated for conversion to mm. Some measurements were made using a Zeiss AxioCam ICc 5 digital camera and ZEN 2 software (Blue edition, 2011). The optical microscopes used were a Zeiss Discovery V8 dissecting microscope, Nikon Labophot compound microscope, and Olympus BX51 compound microscope. Some imagery was obtained using a Leica Z16 APO lens, JVC KY-F75U digital camera, and Synchroscopy Auto-Montage v. 5.01.0005 software. Finally, specimens were mounted in Euparol or Canada balsam as permanent vouchers deposited at the Florida State Collection of Arthropods (FSCA) in Gainesville.

Sample sizes from which data were derived are provided with each species description. Not all character states could be observed nor was it possible to make all measurements on each specimen, as some were prepared for SEM examination and others were slide-mounted with varying success.

The length and width data presented for spiracles are based on cleared, slide-mounted specimens only. The range of measurement made on the same structures as seen under the SEM often do not overlap. We consider the measurements made on slide-mounted specimens to be more accurate, as it is much easier using this preparation to determine whether or not the structure is lying flat when measured.

The data from these descriptions will be incorporated into an interactive system based on that of Carroll et al. (2004) to improve the accuracy of fruit fly larval identifications.

## Descriptions

***Ceratitis rosa*** (partial text of Carroll (1998) in italics; new text and data based on observations of all additional populations in this study combined are shown in parentheses and bold, standard font)

*Diagnosis of third instar*.

*Medium-sized muscidiform larvae with* **(mandibular tooth ventrally grooved), (usually)** *with minute subapical mandibular tooth: usually with 9-11 (rarely 8 or 12) oral ridges; accessory plates* **(present or)** *absent; leaf-like secondary stomal lobes present, sclerotized stomal guards absent; dorsal spinules present on segments T1-A1* **(T1-T2 only; not T3, A1)**; *anterior spiracles usually with 9-10 (rarely 7- 8 or 11-12)* **(usually 10-13, rarely 8 or 15)** *tubules in a single straight* **(to slightly curved or sinuous)** row; base of anterior spiracle cylindrical, **(ca.)** *half as wide as apical width; posterior spiracles with rimae 2.75-3.8 times as wide as long; spiracular processes mostly unbranched* **(to mostly branched, bases narrow to wide)**; *caudal ridge present; anal lobes entire* **(or grooved, posterior lobe often larger than anterior lobe)**.

*Description of third instar*.

*Length 7.7–9.6 mm* **(newly molted 3^rd^ instars estimated at ca. 3.5–4.0 mm)**; *creamy-white, subcylindrical, tapering gradually to cephalic segment*.

**Figure 1.**
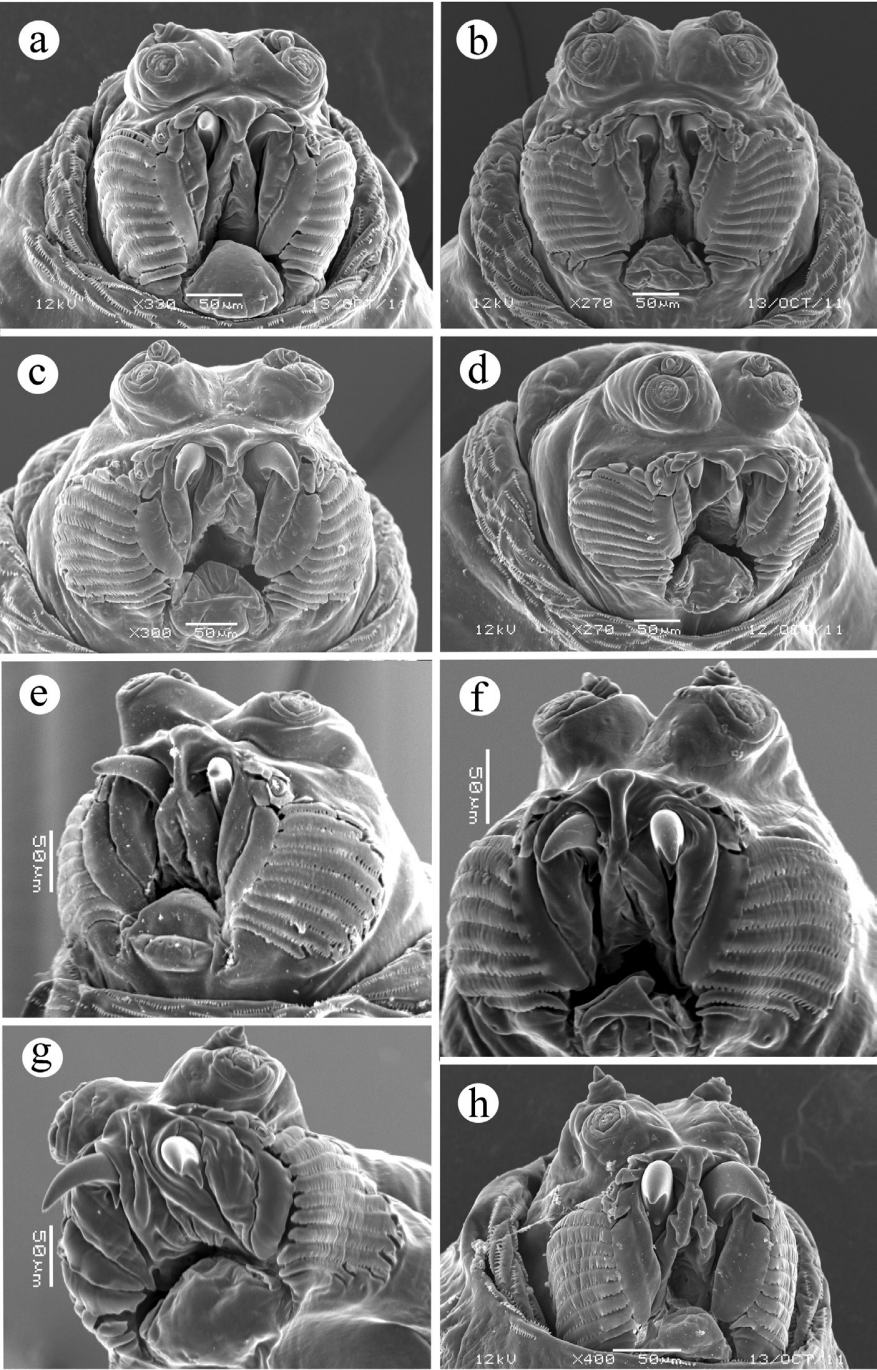
Head, ventral view. **a** *Ceratitis fasciventris* **b** *Ceratitis anonae* **c** *Ceratitis rosa* R1, Kenya **d** *Ceratitis rosa* R2, Kenya **e** *Ceratitis rosa* R1, S. Africa, Nelspruit **f** *Ceratitis rosa* R2, S. Africa, Pretoria **g** *Ceratitis rosa* R2, S. Africa, Stellenbosch **h** *Ceratitis capitata*, Hawaii.

**Figure 2.**
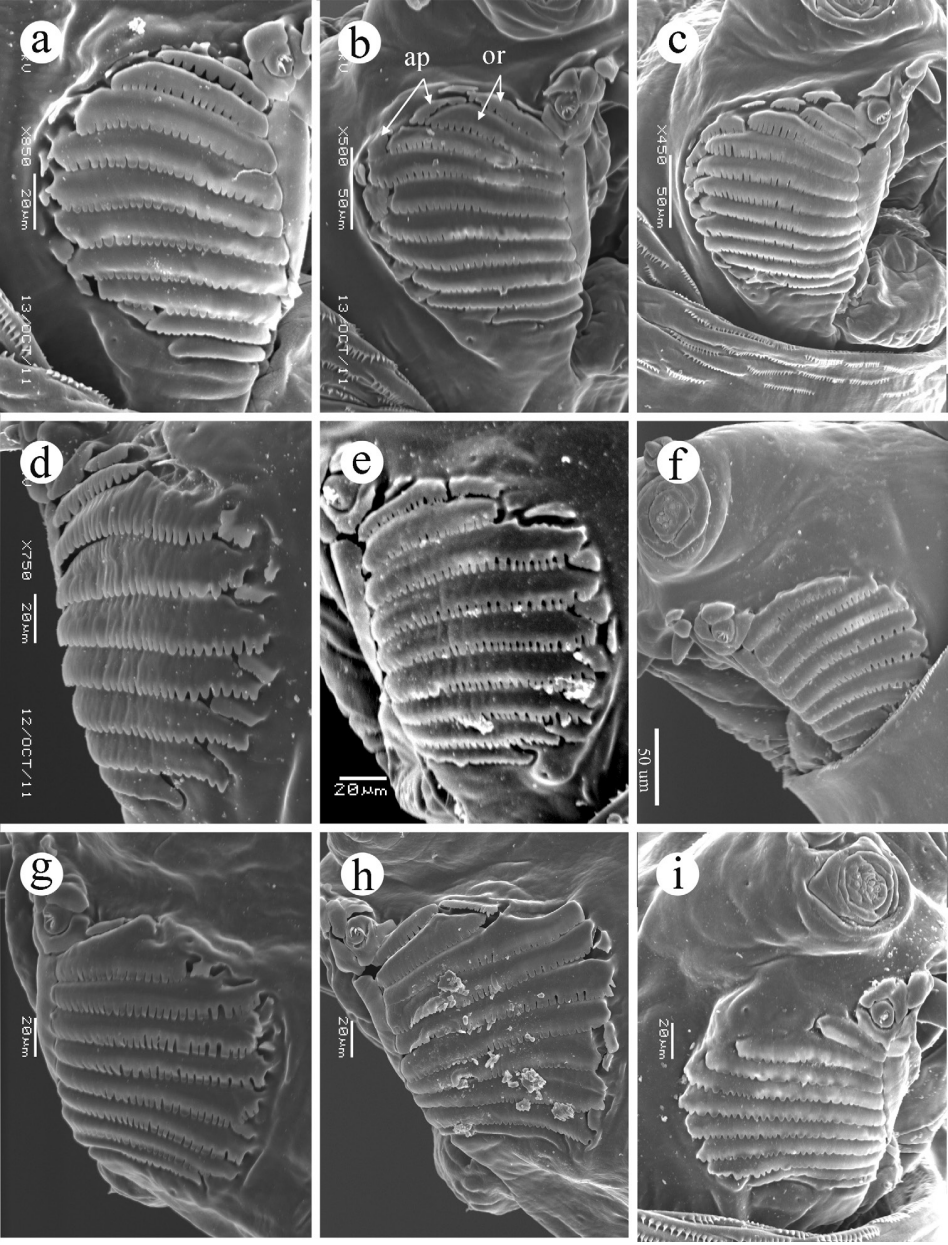
Oral ridges, third instar. **a** *Ceratitis fasciventris* **b** *Ceratitis anonae* **c** *Ceratitis rosa* R1, Kenya **d** *Ceratitis rosa* R2, Kenya **e** *Ceratitis rosa* R1, S. Africa, Nelspruit **f** *Ceratitis rosa* R2, S. Africa, Pretoria **g** *Ceratitis rosa* R2, S. Africa, Stellenbosch **h** *Ceratitis rosa* R2, S. Africa, Stellenbosch **i** *Ceratitis capitata*, Guatemala. Abbreviations: **ap** accessory plate, **or** oral ridge.

*Head* **(Figure 1c–g)** : *with cephalic lobes moderately developed, in lateral view more rounded and protuberant than in Ceratitis capitata* **(true for some *Ceratitis capitata* samples, but in others the difference is very subtle at best; observed differences may be due, at least in part, to the method used to kill and preserve larvae)**; *antenna 2-segmented, both segments with sclerotized walls, the distal segment apically thin-walled and conical; maxillary palp with... sensilla... visible by SEM as 3 papilla sensilla and 2 knob sensilla, the remainder as pits; dorsolateral group of sensilla with 2 papilla sensilla and a pit sensillum, adjacent to but distinct from palp; stomal organ* **(Figure 1c–g)** *with primary lobe small, bearing 3-4 unbranched peg sensilla,... ; 6 secondary lobes present: a broad, flat subtending lobe and a lobe medial to it; usually 2 additional lobes immediately surrounding the primary lobe anteriorly, and usually 2 lobes anteromedial to these, all with edges entire; none of these secondary lobes is strikingly similar to oral ridges; sclerotized stomal guards absent; labium short, triangular, with narrow lateral lobes;... . Oral ridges* **(Figure 2c–h)** *usually 9-11 (but two had 8 and three had 12 ridges on one side only)* **(same range)**; *well developed with margins scalloped to 1/4–1/5 of their depth (visible by SEM), located on a semicircular region laterad of mandible; accessory plates (supernumerary ridges) and other reticulation absent* **(accessory plates present or absent, variably developed in different populations) (Figure 2c–h)**.

**Figure 3.**
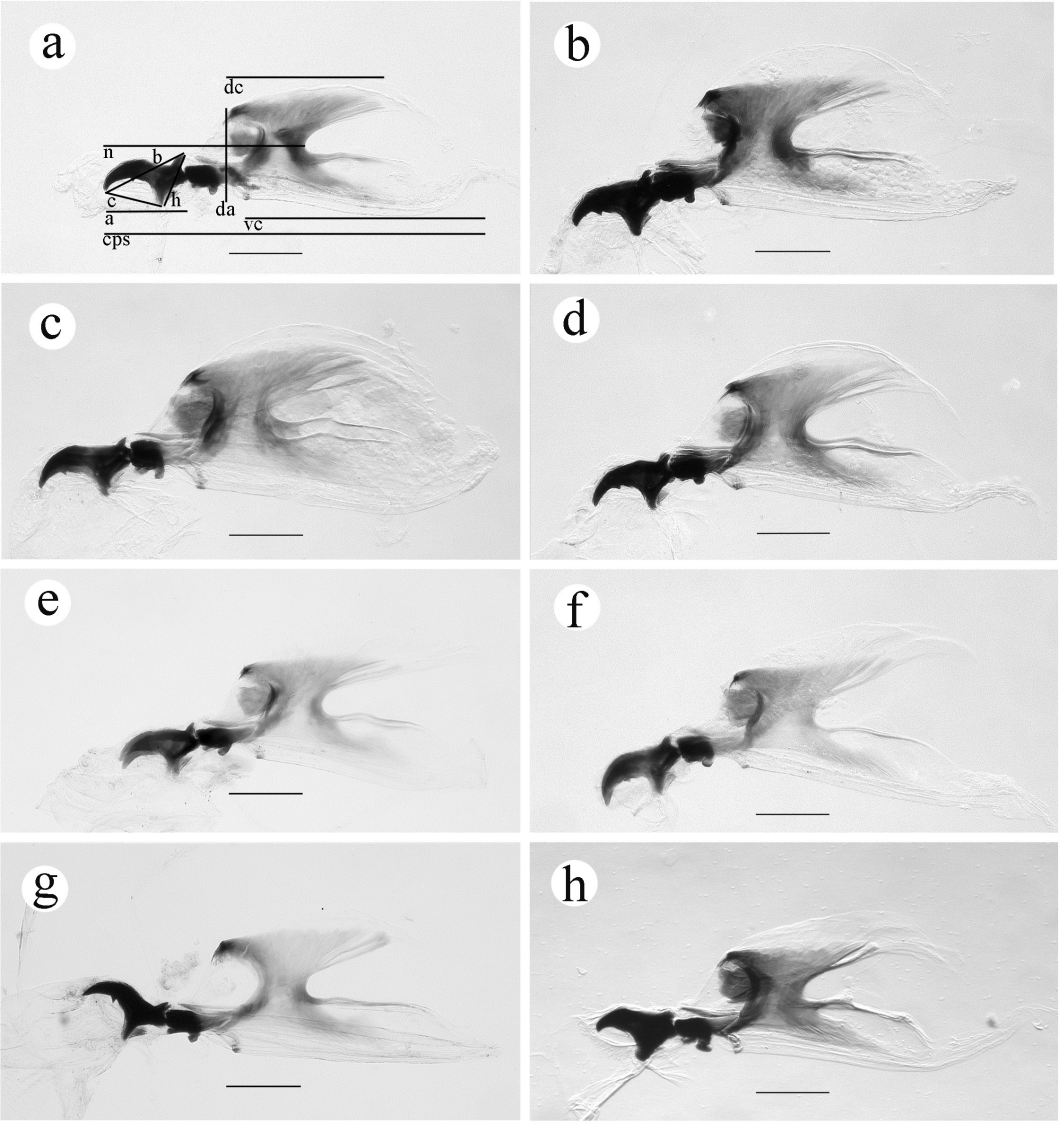
Cephalopharyngeal skeleton. **a** *Ceratitis fasciventris* **b** *Ceratitis anonae* **c** *Ceratitis rosa* R1, Kenya **d** *Ceratitis rosa* R2, Kenya **e** *Ceratitis rosa* R1, S. Africa, Nelspruit **f** *Ceratitis rosa* R2, S. Africa, Pretoria **g** *Ceratitis rosa* R2, S. Africa, Stellenbosch **h** *Ceratitis capitata*, Hawaii. Scale bars 0.20 mm (**a–h**). Abbreviations: **a** mandible tip to posterior prominence, **b** mandible tip to dorsal prominence, **c** mandible tip to ventral prominence, **cps** total length, **da** dorsal arch, **dc** dorsal cornu, **h** mandible height, **n** mandible tip to notch, **vc** ventral cornu.

**Figure 4.**
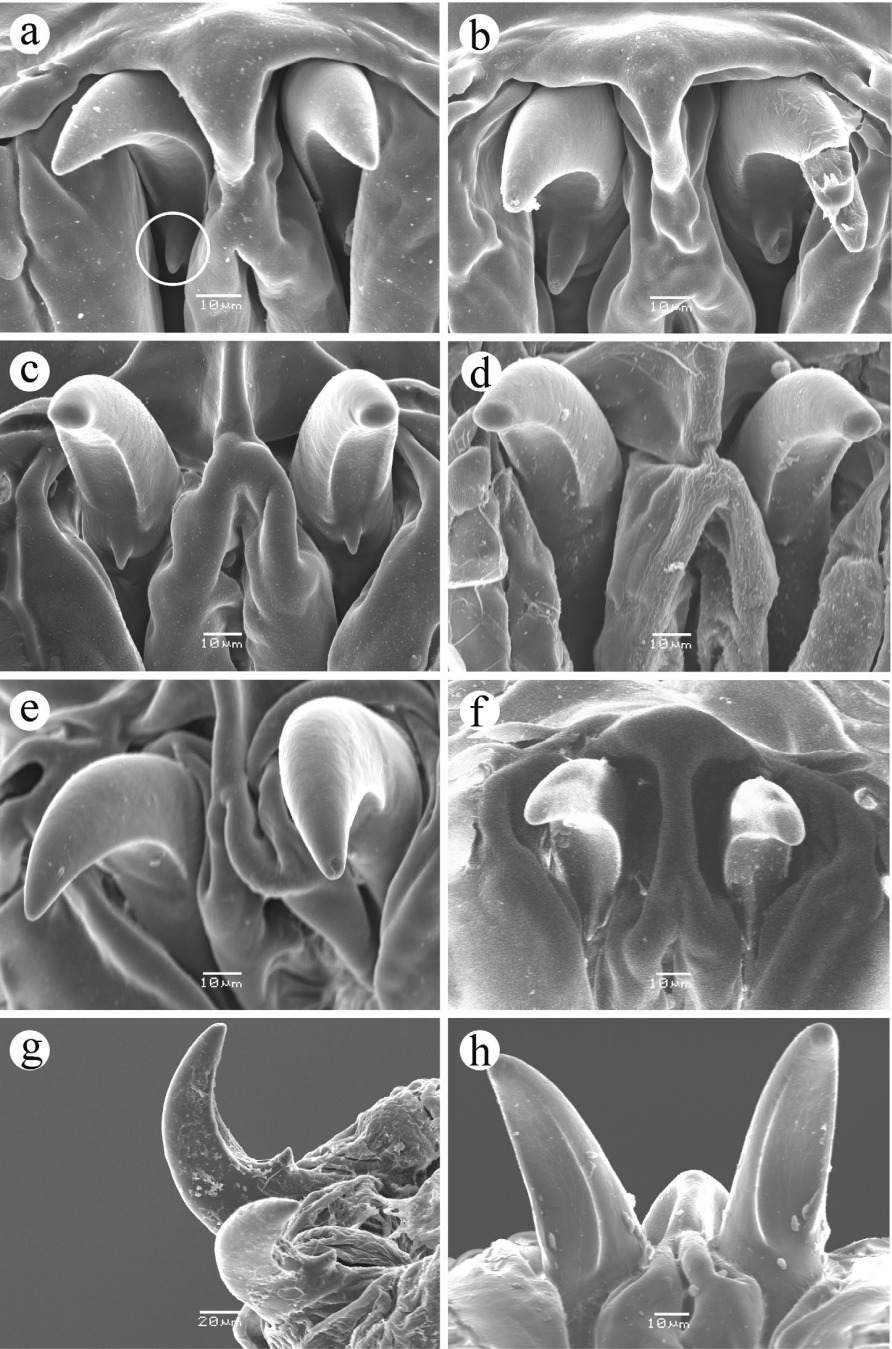
Mouthhooks. **a** *Ceratitis fasciventris* (secondary tooth circled) **b** *Ceratitis anonae* **c** *Ceratitis rosa* R2, Kenya **d** *Ceratitis rosa* R2, Kenya **e** *Ceratitis capitata*, Hawaii **f** *Ceratitis capitata*, Hawaii **g** *Ceratitis capitata*, ex habaneros peppers via Netherlands **h** *Ceratitis capitata*, Guatemala.

*Cephalopharyngeal skeleton* **(CPS) (Figure 3c–g)** *well developed; mandible black to dark brown, apical tooth pointed, with a small subapical tooth (visible in slide-mounted material)* **(present or absent, mandible ventral surface smooth and concave between tip and subapical tooth (Figure 4c–d)**; *hypopharyngeal sclerite black in anterior half, bridge and posterior processes brown; tentoropharyngeal sclerite with dorsal and ventral cornua broadly joined, with strongly pigmented anterior and* **(unpigmented)** *posterior margins, becoming less pigmented dorsally and ventrally; dorsal cornu split posteriorly; ventral cornu with a slight hump midway along dorsal margin, pigmented along dorsal margin and ventrally to slightly more than half its length, with an incomplete window; parastomal sclerite long, stout, brown, slightly hooked* **(straight)** *apically; other sclerites as follows: dental sclerite dark brown, narrow in profile, free from and distinctly posterior to base of mandible; labial sclerites dark brown, slightly shorter than length of hypopharyngeal sclerite bridge, broadly connected to one another to form a pale W-shaped or quadrate sclerite; epipharyngeal sclerite small, faintly pigmented, amorphous* **(not observed)**; *anterior sclerite... present in mature larvae; pharyngeal filter present, with 7* **(not counted)** *lamellate ridges extending the length of the pharynx.* **CPS length 0.99–1.34 mm, mandible tip to notch 0.55–0.75 mm, dorsal cornu length 0.35–0.50, ventral cornu length 0.67–0.95 mm; mandible length a 0.21–0.26 mm, mandible length b 0.22–0.27 mm, mandible length c 0.14–0.19 mm, mandible height 0.15–0.18 mm; hypopharyngeal sclerite length 0.16–0.22 mm, dorsal arch height 0.21–0.33 mm.**

**Figure 5.**
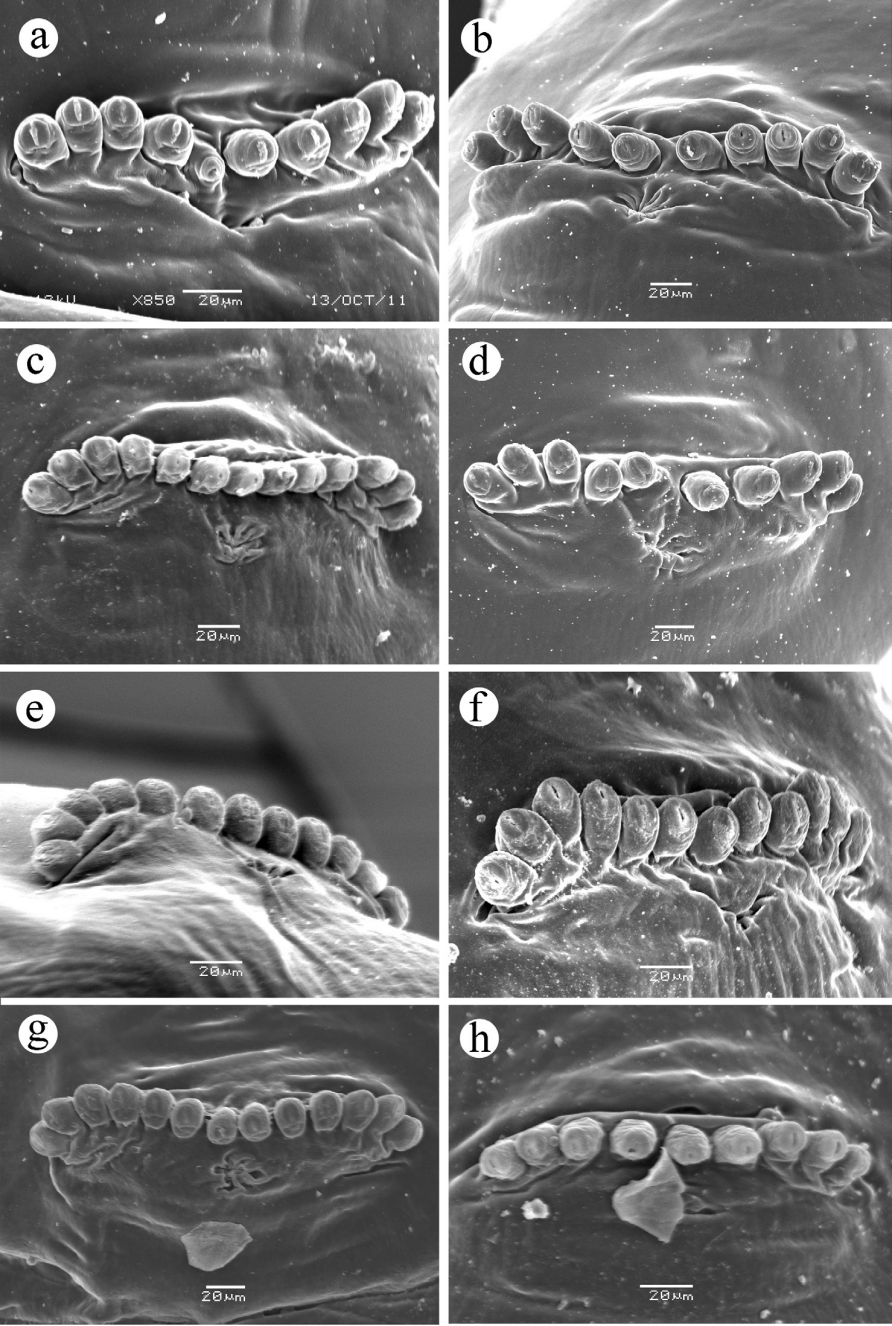
Anterior spiracles. **a** *Ceratitis fasciventris* **b** *Ceratitis anonae* **c** *Ceratitis rosa* R1, Kenya **d** *Ceratitis rosa* R2, Kenya **e** *Ceratitis rosa* R1, S. Africa, Nelspruit **f** *Ceratitis rosa* R2, S. Africa, Pretoria **g** *Ceratitis rosa* R2, S. Africa, Stellenbosch **h** *Ceratitis capitata*, Guatemala.

**Figure 6.**
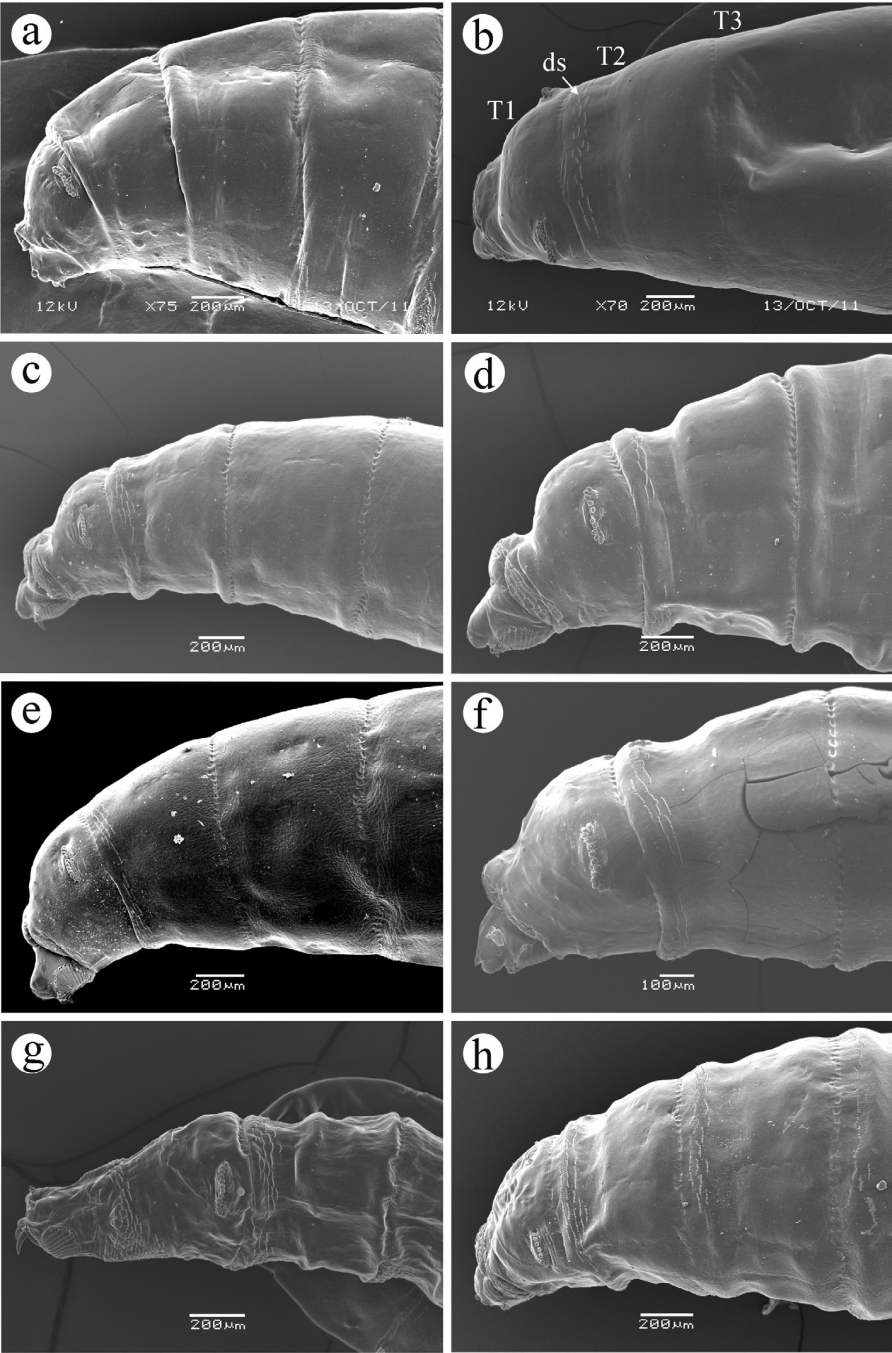
Segments T1-T3. **a** *Ceratitis fasciventris* **b** *Ceratitis anonae* **c** *Ceratitis rosa* R1, Kenya **d** *Ceratitis rosa* R2, Kenya **e** *Ceratitis rosa* R1, S. Africa, Nelspruit **f** *Ceratitis rosa* R2, S. Africa, Pretoria **g** *Ceratitis rosa* R2, S. Africa, Stellenbosch **h** *Ceratitis capitata*, Guatemala. Abbreviations: **T1**, **T2**, **T3** thoracic segments 1 to 3, **ds** dorsal spinules.

*Anterior spiracle* **(Figure 5c–g)** *pale golden brown, projecting, usually with 8-10* **(10-13)** *tubules (but one had 7 and one had 11 on one side only; one had 11 on both sides)* **(rarely 8 or 15)** *closely spaced in a single straight* **(to curved or sinuous)** *row; distal width 0.145—0.166 mm (n = 4)* **(0.16–0.24 mm, n = 18)**, *base cylindrical, about half as wide as distal width; tubules about as long as wide, rounded apically, each with a slitlike opening; felt chamber as in Figure 10; unpigmented ecdysial scar posterior to tubules. Segments T1-T3 and usually A1* **(T1-T2, but not T3 and A1) (Figure 6c–g)** *with broken rows of weak, conical spinules on dorsal anterior margin, with 3-5, 3-5, 1-4* **(0)**, *and 1-2* **(0)** *rows of spinules, respectively, at dorsal midline; on Tl and T2 the spinulose area encircles the body* **(T1 only)**, *while on T3* **(T2)** *the ventral spinulose area is separated from that of the dorsum; dorsal spinules absent on A2-A8* **(T3-A8)**; *ventral spinulose areas on Tl with 10-12 rows, T2 and T3 with 3-7 rows each, and Al with 4-7 rows of posteriorly directed spinules; ventrally, segments A2 - A7 with 9-11 rows and A8 with 6-9 rows of spinules that are alternately arranged in groups of anteriorly and posteriorly directed rows, typically arranged as follows: 1-3 rows of small (approximately 0.005 mm long), anteriorly directed spinules that appear to overlap segmental lines and actually pertain to the preceding segment; 2-4 rows of small, posteriorly directed spinules; 1-2 rows of anteriorly directed spinules, and 2- 5 rows of posteriorly directed spinules, some of which may reach 0.014 mm in length*.

**Figure 7.**
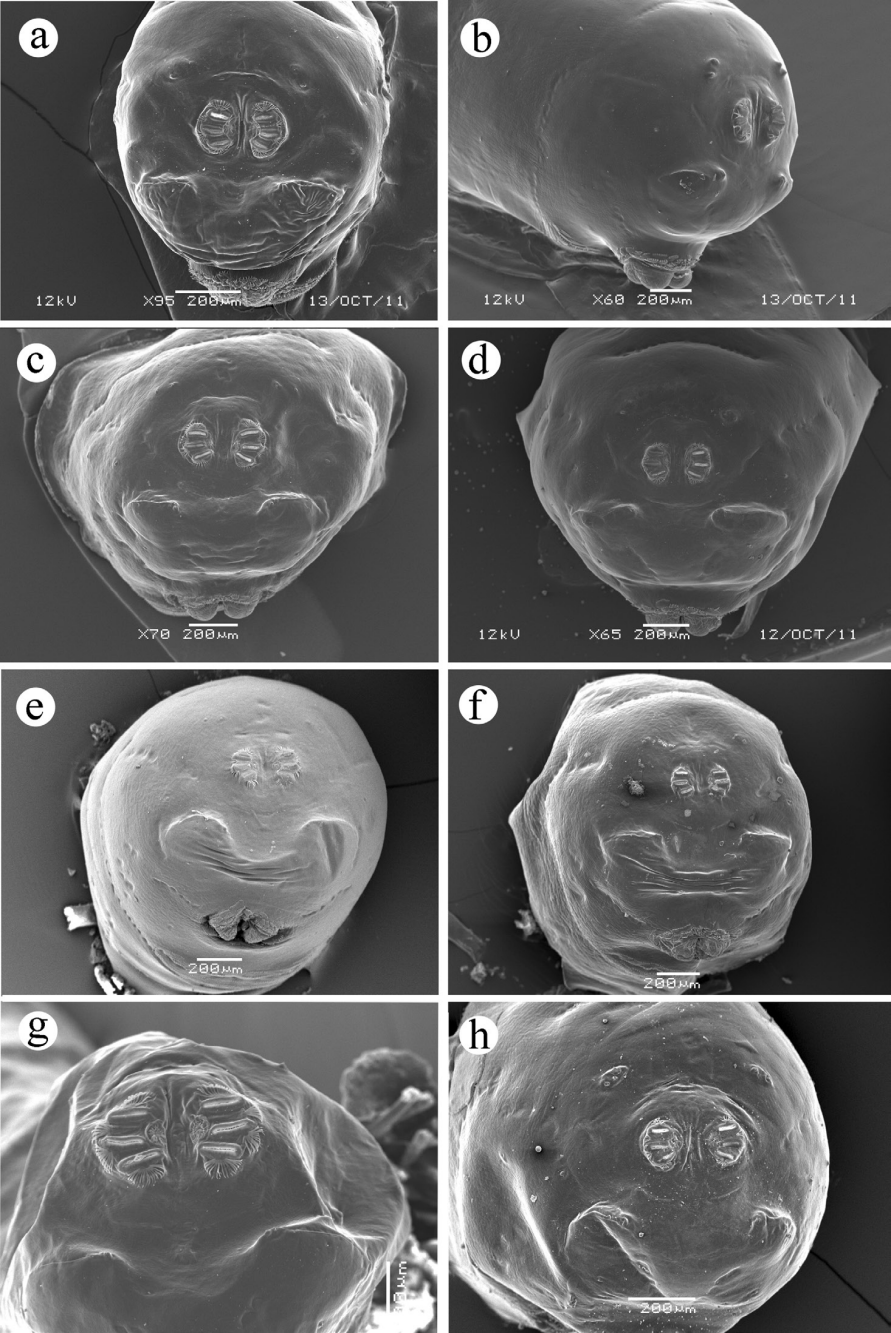
Caudal segment. **a** *Ceratitis fasciventris* **b** *Ceratitis anonae* **c** *Ceratitis rosa* R1, Kenya **d** *Ceratitis rosa* R2, Kenya **e** *Ceratitis rosa* R1, S. Africa, Nelspruit **f** *Ceratitis rosa* R2, S. Africa, Pretoria **g** *Ceratitis rosa* R2, S. Africa, Stellenbosch **h** *Ceratitis capitata*, Kenya.

**Figure 8.**
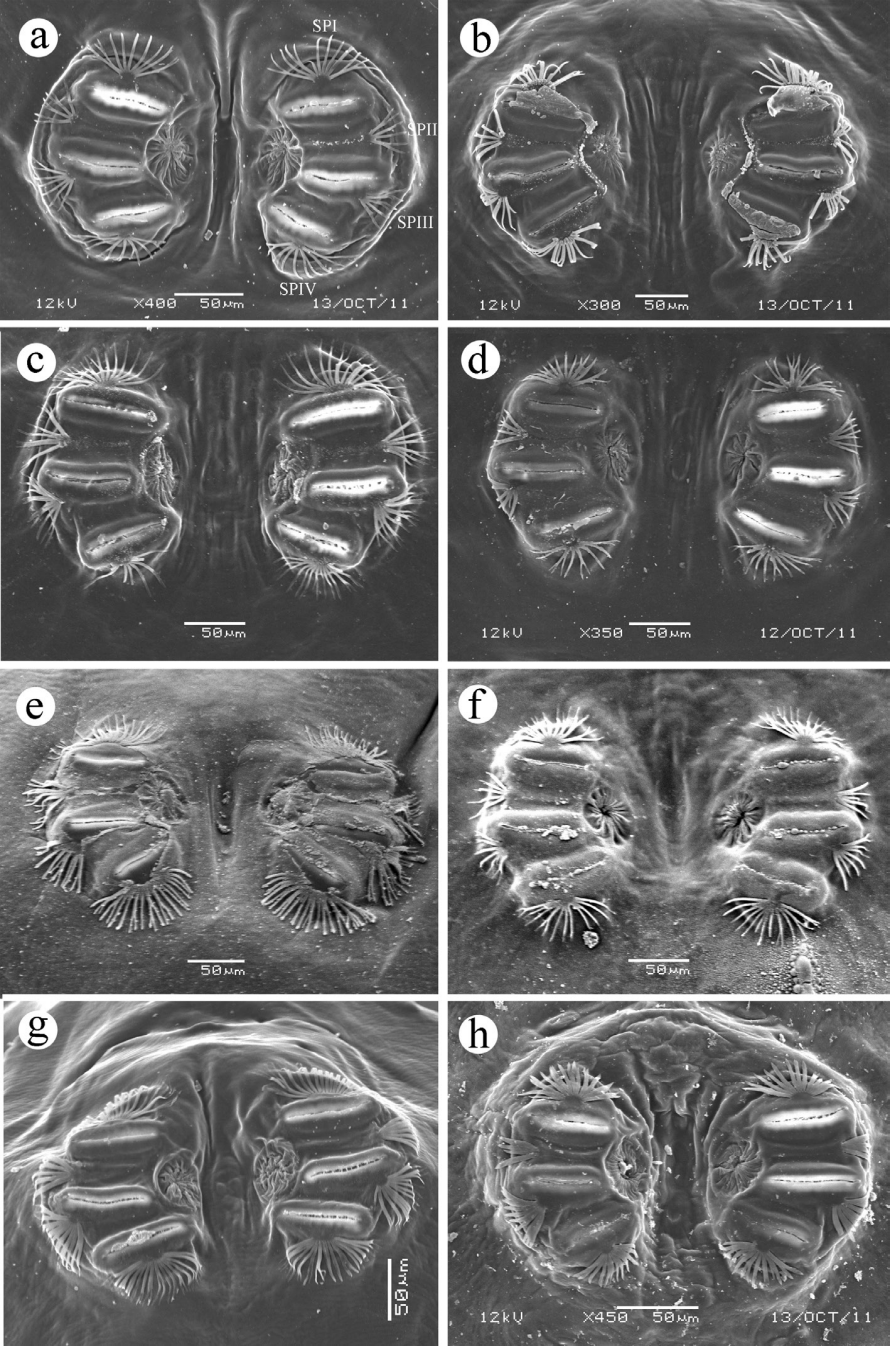
Posterior spiracles. **a** *Ceratitis fasciventris* **b** *Ceratitis anonae* **c** *Ceratitis rosa* R1, Kenya **d** *Ceratitis rosa* R2, Kenya **e** *Ceratitis rosa* R1, S. Africa, Nelspruit **f** *Ceratitis rosa* R2, S. Africa, Pretoria **g** *Ceratitis rosa* R2, S. Africa, Stellenbosch **h** *Ceratitis capitata*, Kenya. Abbreviations: **SPI**, **SPII**, **SPIII**, **SPIV** spiracular processes 1 to 4.

**Figure 9.**
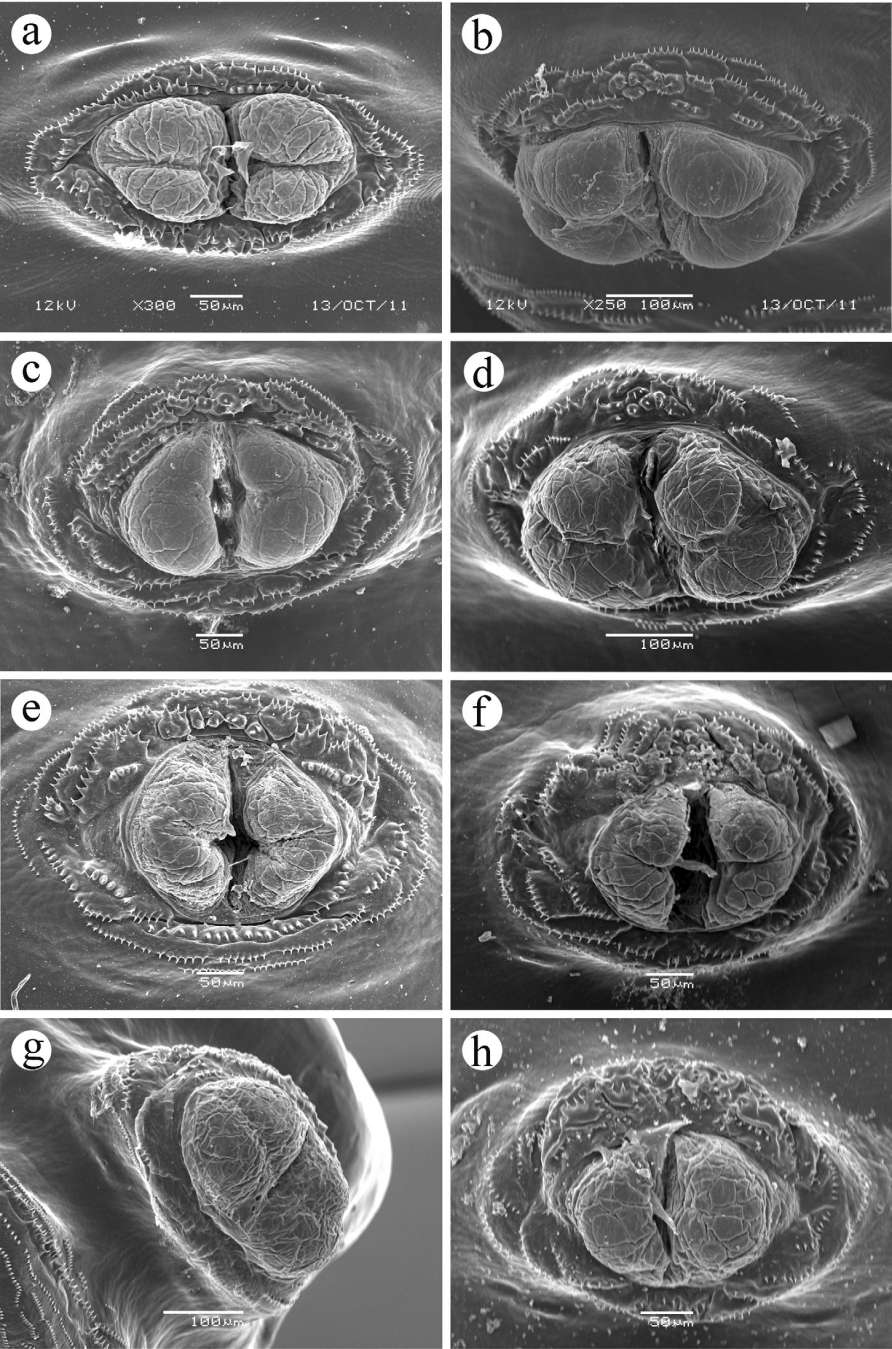
Anal lobes. **a** *Ceratitis fasciventris* **b** *Ceratitis anonae* **c** *Ceratitis rosa* R1, Kenya **d** *Ceratitis rosa* R2, Kenya **e** *Ceratitis rosa* R1, S. Africa, Nelspruit **f** *Ceratitis rosa* R2, S. Africa, Pretoria **g** *Ceratitis rosa* R2, S. Africa, Stellenbosch **h** *Ceratitis capitata*, Kenya.

*Caudal segment* **(Figure 7c–g)** *with a caudal ridge on the intermediate region, without a dark transverse line in the medial region; 10 pairs of sensilla present as follows: dorsal area with Dl and D2 on separate papillae very close to one another; lateral area with I3 and L on separate papillae; intermediate area with intermediate tubercle well developed, bearing the following sensilla: I1a and I1b (on the same papilla or very close together, near medial end of caudal ridge), and I2 (below caudal ridge); ventral area with 3 V sensilla (one as a papilla sensillum and two as pit sensilla). Posterior spiracle* **(Figure 8c–g)** *above midline, with 3 slit-like openings, dorsal and central slit subparallel, ventral slit more medial and at an angle to the other two; rimae about 2.75-3.8 times longer than wide (0.065-0.082* **(0.06–0.09)** *mm long; 0.021–0.025 mm wide), separated from midline by approximately 2-3 times the length of the rima; spiracular processes well-developed, about half as long as rimae, mostly unbranched* **(to mostly branched)**, *some with 1-2 branches; numbers of trunks and tips as follows; I (dorsal) (8-9, 10-12)* **(7-24, 11-38)**, *II (3, 5-6)* **(3-7, 5-15)**, *III (4-7, 7-11)* **(3-13, 5-21)**, *IV (ventral) (9-10, 11-14)* **(4-17, 8-25)**. *Anal lobes* **(Figure 9c–g)** *well-developed, protruding, entire (rarely grooved)* **(usually grooved, posterior lobe often larger than anterior lobe)**, *surrounded by 2-4 broken rows of spinules*.

*Specimens examined.—SOUTH AFRICA: ex culture, Brian Barnes (n = 40, USNM, TAMU)* [Specimens described by Carroll (1998) came from a laboratory colony that originated with material from Stellenbosch, Western Cape, ex guava, ca. 1972-1973; Brian Barnes, personal communication, 28 Oct 2011. They were not re-examined in this study]. **R1 (hot type): KENYA n = 12 SEM + 5 slide, SOUTH AFRICA Nelspruit n = 5 SEM + 5 slide. R2 (cold type): KENYA n = 15 SEM + 5 slide, SOUTH AFRICA Pretoria n = 5 SEM + 5 slide, SOUTH AFRICA Stellenbosch n = 4 SEM + 5 slide.**

*Variation among populations.* There is considerable variation among populations in various quantitative characters, and some ranges do not overlap. See accompanying tables of comparative data for various measures and counts taken on the oral ridges, accessory plates, stomal organ, and anterior spiracles (Table 1), cephalopharyngeal skeleton (Table 2; mandible abbreviated as MH), posterior spiracles and anal lobes (Table 3), and mandible secondary tooth (Table 4).

**Table 1.** Oral ridges and anterior spiracle quantitative data (length in mm).

Species	no. Oral Ridges	no. Accessory Plates	Stomal Organ: no. petals	Anterior spiracle: # tubules	Anterior spiracle: apical width	Anterior spiracle: tracheal width
*rosa* R1 Kenya	8–12	7-11, well-developed, numerous serrate	2-3 subtending + 2-3 medial	10–12	0.17–0.21	0.10–0.12
*rosa* R2 Kenya	8–11	4-7, well-developed, some serrate	3-4 subtending + 2-3 medial	10–11	0.18–0.22	0.10–0.12
*rosa* R1 S.A. Nelspruit	9–12	7-8, well-developed, some serrate	2-4 subtending + 2-3 medial	8–15	0.16–0.21	0.08–0.10
*rosa* R2 S.A. Pretoria	8–10(+)	0-3 nubs or short linear	4 subtending + 2 medial	11–13	0.19–0.21	0.08–0.10
*rosa* R2 S.A. Stellenbosch	8–10	0-1 serrate + 4-7 nubs	3-4 subtending + 2-3 medial	12–13	0.19–0.24	0.09–0.11
*anonae*	10–11	8-12, well-developed, some serrate	4 subtending + 2 medial	10–13	0.19–0.24	0.10–0.12
*fasciventris*	10	3-8, small or nubs	3-4 subtending + 1-2 medial	9–12	0.14–0.17	0.09–0.12
*capitata*	8–12	absent	1-4 subtending + 1-2 medial	9–12	0.16–0.19	0.06–0.12

**Table 2.** Cephalopharyngeal skeleton quantitative data (length in mm).

Species	CPS total length	MH tip to notch	dorsal cornu length	ventral cornu length	MH secondary tooth	length (a) MH tip to posterior prominence	length (b) MH tip to dorsal prominence	length (c) MH tip to ventral prominence	height MH dorsal prominence to ventral prominence	Hypopharyngeal sclerite length	dorsal arch
*rosa* R1 Kenya	1.16–1.23	0.61–0.65	0.44–0.45	0.77–0.87	present	0.24–0.25	0.25–0.25	0.16–0.17	0.15–0.16	0.16–0.19	0.30–0.33
*rosa* R2 Kenya	1.15–1.27	0.60–0.75	0.35–0.49	0.72–0.81	present/absent	0.21–0.25	0.22–0.26	0.16–0.16	0.16–0.18	0.16–0.17	0.29–0.33
*rosa* R1 Nelspruit	0.99–1.26	0.55–0.60	0.35–0.44	0.67–0.88	present	0.21–0.25	0.23–0.25	0.14–0.16	0.15–0.16	0.16–0.20	0.21–0.28
*rosa* R2 Pretoria	1.16–1.34	0.59–0.60	0.40–0.47	0.75–0.95	present	0.22–0.25	0.24–0.27	0.14–0.16	0.15–0.18	0.18–0.22	0.25–0.26
*rosa* R2 Stellenb.	1.14–1.20	0.60–0.64	0.44–0.50	0.73–0.78	present	0.24–0.26	0.23–0.26	0.16–0.19	0.15–0.17	0.16–0.18	0.30–0.31
*anonae*	1.16–1.23	0.59–0.63	0.43–0.44	0.75–0.80	present	0.20–0.25	0.25–0.26	0.16–0.16	0.16–0.17	0.16–0.18	0.29–0.31
*fasciventris*	0.89–1.14	0.52–0.60	0.37–0.43	0.52–0.72	present	0.21–0.23	0.21–0.25	0.15–0.16	0.14–0.16	0.12–0.17	0.25–0.30
*capitata*	1.06–1.11	0.53–0.57	0.27–0.43	0.69–0.73	present/absent	0.21–0.23	0.20–0.22	0.14–0.15	0.14–0.16	0.16–0.17	0.25–0.29

**Table 3.** Posterior spiracle quantitative measures and anal lobes (length in mm).

Species	Post spiracle slit length	Post spiracle slit width	Post spiracle tracheal width	SP-I no. trunks / no. tips	SP-I ratio tips/trunks	SP-I basal width	SP-I ratio width/slit length
*rosa* R1 Kenya	0.08–0.09	0.02–0.03	0.16–0.21	10-18 / 14-29	1.0–2.0	0.02–0.04	0.25–0.44
*rosa* R2 Kenya	0.07–0.09	0.02– 0.03	0.17–0.19	7-11 / 11-20	1.2–2.3	0.01–0.02	0.15–0.24
*rosa* R1 S.A. Nelspruit	0.06–0.08	0.02– 0.03	0.14–0.17	13-14 / 13-24	1.0–1.7	0.02–0.04	0.27–0.52
*rosa* R2 S.A. Pretoria	0.07–0.07	0.02– 0.03	0.12–0.16	8-14 / 13-20	1.2–1.8	0.01–0.02	0.28–0.30
*rosa* R2 S.A. Stellenb.	0.08–0.09	0.02– 0.03	0.15–0.17	12-24 / 24-38	1.6–2.1	0.04–0.05	0.34–0.63
*anonae*	0.08–0.09	0.02– 0.03	0.18–0.21	9-12 / 13-21	1.3–1.9	0.02– 0.04	0.22–0.32
*fasciventris*	0.07–0.07	0.02– 0.03	0.15–0.18	2-9 / 3-14	1.5–2.2	0.01–0.02	0.07–0.28
*capitata*	0.07–0.08	0.02– 0.03	0.16–0.21	6-15 / 10-17	1.1–1.7	0.01–0.02	0.17–0.33
	**SP-II no. trunks / no. tips**	**SP-III no. trunks / no. tips**	**SP-IV no. trunks / no. tips**	**SP-IV ratio tips/trunks**	**SP-IV basal width**	**SP-IV ratio width/slit length**	**Anal lobe shape**
*rosa* R1 Kenya	4-7 / 7-12	6-13 / 7-20	6-14 / 11-23	1.3–2.1	0.02–0.04	0.18–0.41	grooved
*rosa* R2 Kenya	3-5 / 5-8	3-6 / 5-9	4-8 / 8-16	1.2–3.2	0.01–0.02	0.11–0.20	grooved
*rosa* R1 S.A. Nelspruit	4-6 / 7-12	6-8 / 9-15	10-15 / 10-24	1.0–1.9	0.02–0.03	0.19–0.49	grooved
*rosa* R2 S.A. Pretoria	3-6 / 5-8	3- 9 / 6-11	8-12 / 8-12	1.2–1.7	0.01–0.03	0.13–0.38	grooved
*rosa* R2 S.A. Stellenb.	4-7 / 9-15	6-12 / 10-21	10-17 / 18-25	1.5–2.1	0.03–0.04	0.24–0.42	grooved
*anonae*	3-6 / 6-9	4-8 / 5-11	6-11 / 11-16	1.2–2.2	0.01–0.03	0.15–0.27	grooved
*fasciventris*	2-4 / 4-7	2-6 / 4-9	4-8 / 6-12	1.5–2.0	0.01–0.01	0.07–0.19	grooved
*capitata*	3-4 / 4-5	2-6 / 4-11	2-10 / 3-14	1.2–1.5	0.01–0.01	0.17–0.17	entire to grooved

**Table 4.** Development of mandible secondary tooth.

	*rosa* R2 Kenya	*rosa* R1 Kenya	*rosa* R1 Nelspruit	*rosa* R2 Pretoria	*rosa* R2 Stellenbosch	*capitata* Kenya	*capitata* Hawaii	*capitata* Guatemala	*capitata* E2004-6626	*anonae*	*fasciventris*
Well developed	6	6	2	2	8	4	2	0	4	7	8
Poorly developed	2	0	0	0	0	1	5	6	0	0	0
Present/Absent	2	0	0	0	0	0	0	0	0	1	0
Absent	1	0	0	0	0	8	6	3	0	0	0
N =	11	6	2	2	8	13	13	9	4	8	8

Some notable differences among populations include:

Dorsal spinules–Carroll (1998) described a Stellenbosch-derived colony as having dorsal spinules present on segments T1-T3 and usually A1. In all populations observed in this study, however, including a newly derived colony from Stellenbosch, larvae have dorsal spinules present on T1 and T2 only, but none on T3, A1 or beyond.

Oral ridge accessory plates–Carroll (1998) described a Stellenbosch-derived colony as lacking accessory plates. However, among populations observed here, larvae of R1-Kenya, R2-Kenya, and R1-Nelspruit have accessory plates present, numerous and well-developed to the point of having serrate edges. Alternatively, larvae of R2-Pretoria have accessory plates lacking or minimally present as a few thin ridges or nubs, without serrate edges, and on R2-Stellenbosch they range from a single well-developed serrate accessory plate plus a few additional nubs, to numerous nubs, to completely lacking.

Anal lobes–Carroll (1998) described a Stellenbosch-derived colony as having anal lobes entire (rarely grooved). In all populations observed in this study, however, including a newly derived colony from Stellenbosch, larvae have grooved anal lobes.

Quantitative measures that do not (or minimally) overlap among samples include the trachea diameter at base of anterior spiracle (larger in Kenyan populations than South African populations regardless of hot or cold type); length from tip of mandible to notch in CPS, length from tip of mandible to tip of ventral prominence, and height of dorsal arch (larger in R1- and R2-Kenya + R2-Stellenbosch vs. smaller in R1-Nelspruit and R2-Pretoria). Various other individual pairings of samples do not overlap for some measures and counts especially those associated with the posterior spiracles (See Table 3). These differences in quantitative characters may merely reflect relatively small sample sizes or the result of artificial selection in laboratory colonies.

### Ceratitis anonae

Taxon classificationAnimaliaDipteraTephritidae

Figures 1b , 2b , 3b , 4b , 5b , 6b , 7b , 8b , 9b

#### Diagnosis of third instar.

Medium-sized muscidiform larvae with mandibular tooth ventrally grooved, with minute subapical mandibular tooth; with 10-11 oral ridges; accessory plates present; petal-like secondary stomal lobes present, sclerotized stomal guards absent; dorsal spinules present on segments T1-T2; anterior spiracles with 10-13 tubules in a single straight to slightly curved or sinuous row; base of anterior spiracle cylindrical, ca. half as wide as apical width; posterior spiracles with rimae ca. 3 times longer than wide; spiracular processes mostly unbranched to mostly branched with narrow bases; caudal ridge present; anal lobes grooved, posterior portion often larger than anterior portion.

#### Description of third instar

(differences from *Ceratitis rosa* description above are noted in bold font). Similar to *Ceratitis rosa*, except length **3.8–8.9 mm**; oral ridges **10–11**; accessory plates **present, well-developed**; parastomal sclerite **straight to slightly curved**, **not hooked apically**; **CPS length 1.16–1.23 mm, mandible secondary tooth present, mandible tip to notch 0.59–0.63 mm, dorsal cornu length 0.43–0.44, ventral cornu length 0.75–0.80 mm; mandible length a 0.20–0.25 mm, mandible length b 0.25–0.26 mm, mandible length c ca. 0.16 mm, mandible height 0.16–0.17 mm; hypopharyngeal sclerite length 0.16–0.18 mm, dorsal arch height 0.29–0.31 mm.** Anterior spiracle with **10–13** tubules, closely spaced in a single **slightly sinuous** row, apical width **0.19–0.24 mm (n = 7)**; segments **T1-T2** with broken rows of weak, conical spinules on dorsal anterior margin; on **T1** the spinulose area encircles the body, while on **T2** the ventral spinulose area is separated from that of the dorsum; dorsal spinules **absent on T3-A8**; posterior spiracle rimae **0.08–0.09** mm long, ca. 0.025 mm wide; spiracular processes **mostly unbranched to mostly branched, base of SP-I and SP-IV narrow**, numbers of trunks and tips as follows; I (dorsal) (**9-12, 13-21**), II (**3-6, 6-9**), III (**4-8, 5-11**), IV (ventral) (**6-11, 11-16**); anal lobes **grooved, posterior portion often larger than anterior portion**.

#### Specimens examined.

n = 4 (SEM) + 5 (slide).

### Ceratitis fasciventris

Taxon classificationAnimaliaDipteraTephritidae

Figures 1a , 2a , 3a , 4a , 5a , 6a , 7a , 8a , 9a

#### Diagnosis of third instar.

Medium-sized muscidiform larvae with mandibular tooth ventrally grooved, with minute subapical mandibular tooth; usually with 10 oral ridges; accessory plates weakly developed; petal-like secondary stomal lobes present; sclerotized stomal guards absent; dorsal spinules present on segments T1-T2; anterior spiracles with 9-12 tubules in a single sinuous row; base of anterior spiracle cylindrical, ca. half as wide as apical width; posterior spiracles with rimae ca. 3 times longer than wide; spiracular processes mostly unbranched to mostly branched, with narrow bases; caudal ridge present; anal lobes grooved, lobes subequal or posterior lobe larger.

#### Description of third instar

(differences from *Ceratitis rosa* description above are noted in bold font). Similar to *Ceratitis rosa*, except length **3.2–6.8 mm**; oral ridges usually **10**; accessory plates **weakly developed**; parastomal sclerite straight to curved, not hooked apically; anterior sclerite **present or absent**; **CPS length 0.89–1.14 mm, mandible secondary tooth present, mandible tip to notch 0.52–0.60 mm, dorsal cornu** **length 0.37–0.43 mm, ventral cornu length 0.52–0.72 mm; mandible length a 0.21–0.23 mm, mandible length b 0.21–0.25 mm, mandible length c 0.15–0.16 mm, mandible height 0.14–0.16 mm; hypopharyngeal sclerite length 0.12–0.17 mm, dorsal arch height 0.25–0.30 mm**; anterior spiracle with **9–12** tubules, tubules closely spaced in a single **slightly sinuous** row; distal width **0.14–0.17 mm (n = 5)**; segments **T1-T2** with broken rows of weak, conical spinules on dorsal anterior margin; on **T1** the spinulose area encircles the body, while on **T2** the ventral spinulose area is separated from that of the dorsum; dorsal spinules **absent on T3-A8**; posterior spiracle rimae **ca. 0.07** mm long; spiracular processes mostly unbranched to mostly branched, base of SP-I and SP-IV narrow, numbers of trunks and tips as follows; I (dorsal) (**2-9, 3-14**), II (**2-4, 4-7**), III (**2-6, 4-9**), IV (ventral) **(4-8, 6-12**). Anal lobes **grooved**, lobes subequal or posterior lobe larger.

#### Specimens examined.

n = 5 (SEM) + 10 (slide).

### Ceratitis capitata

Taxon classificationAnimaliaDipteraTephritidae

Figures 1h , 2h , 3h , 4e-h , 5h , 6h , 7h , 8h , 9h

#### Diagnosis of third instar.

Medium-sized muscidiform larvae with mandibular tooth ventrally grooved, minute subapical mandibular tooth present or absent; with 8-12 oral ridges; accessory plates absent; petal-like secondary stomal lobes present; sclerotized stomal guards absent; dorsal spinules present on segments T1-T3; anterior spiracles with 9-12 tubules in a single sinuous row; base of anterior spiracle cylindrical, ca. half as wide as apical width; posterior spiracles with rimae ca. 3 times longer than wide; spiracular processes mostly unbranched, with narrow bases; caudal ridge present; anal lobes entire or grooved, lobes subequal.

#### Description of third instar

(differences from *Ceratitis rosa* description above are noted in bold font). Similar to *Ceratitis rosa*, except length **3.9–8.7 mm**; oral ridges **9–11 (rarely 8 or 12)**; accessory plates **absent**; parastomal sclerite straight to curved; **CPS length 1.06–1.11 mm, mandible secondary tooth present or absent, mandible tip to notch 0.53–0.57 mm, dorsal cornu length 0.27–0.43 mm, ventral cornu length 0.69–0.73 mm; mandible length a 0.21–0.23 mm, mandible length b 0.20–0.22 mm, mandible length c 0.14–0.15 mm, mandible height 0.14–0.16 mm; hypopharyngeal sclerite length 0.16–0.17 mm, dorsal arch height 0.25–0.29 mm**; anterior spiracle with **9–12** tubules, tubules closely spaced in a single **slightly sinuous** row; distal width **0.16–0.19 mm (n = 8)**; segments **T1-T3 (rarely A1)** with broken rows of weak, conical spinules on dorsal anterior margin; on **T1** the spinulose area encircles the body, while on **T2-T3** the ventral spinulose area is separated from that of the dorsum; dorsal spinules **absent on A1-A8**; posterior spiracle rimae **0.07–0.08** mm long; spiracular processes mostly unbranched, numbers of trunks and tips as follows: I (dorsal) (**6-15, 10-17**), II (**3-4, 4-5**), III (**2-6, 4-11**), IV (ventral) **(2-10, 3-14**). Anal lobes **entire or grooved**, lobes subequal.

#### Specimens examined.

n = 41 (SEM) + 9 (slide).

## Discussion

Although larval stages of numerous fruit fly species have been described, very few are based on wide geographic sampling, and often they are based on colony material. The extent to which these descriptions reflect actual variation in nature is generally unknown. Specimens from laboratory colonies are probably more homogenous than those collected directly from the wild. Sample sizes used in this study are small, so we have to expect that the range of measurements presented here is less than that in nature.

There are consistent morphological differences in larval character states among some of the *Ceratitis rosa* populations studied here. Some of these would be considered key diagnostic characters to recognize different species in other genera, e.g. *Anastrepha* (Steck et al. 1990). Whether they represent intra-specific variation or diagnostic differences among biologically distinct taxa of *Ceratitis* cannot be answered using these data alone. The larval morphological differences observed among populations examined here are not congruent in any simple way with the R1 and R2 designations. The diagnosis and description of *Ceratitis rosa* *s.l.* given here incorporates data from all of the populations observed. If some of these eventually are determined to represent different taxa, then the diagnosis and description of *Ceratitis rosa* *s.s.* may require alteration, as some character states do not overlap among populations. Additional data of other types (e.g., genetic, behavioral, etc.) are required to determine the taxonomic interpretation of larval morphology data.

Even beyond the question of possible cryptic species among *Ceratitis rosa* *s.l.*, it is maddeningly difficult to find reliable diagnostic differences in larval morphology among species of the FAR complex. Many of the quantitative larval characters seem to be little constrained and their ranges vary wildly. However, *Ceratitis fasciventris* can generally be distinguished from *Ceratitis rosa* *s.l.* and *Ceratitis anonae* by its smaller dimensions of the CPS and anterior spiracle apical width, and lower counts of spiracular processes and narrowness of their bases.

*Ceratitis capitata* larvae can be separated from most individuals of the FAR complex by the absence of oral ridge accessory plates and the presence of dorsal spinules on T3. Also the shape of the anterior spiracle seems consistently different (smoothly expanded from the base to the tubules), as described and illustrated by Carroll (1998). Presence vs. absence of a small secondary tooth on the mandible is not a reliable character state to separate them. While the secondary tooth is typically present and easy to see on FAR larvae, it may be absent or poorly developed as seen in the *Ceratitis rosa* R2 colony at ICIPE. Conversely, the secondary tooth is often present on larvae of *Ceratitis capitata*, although it frequently is poorly developed and not likely to be noticed except by use of SEM.

It should be noted that presence vs. absence of oral ridge accessory plates has been used as a key character to separate larvae of the genus *Ceratitis* from those of *Bactrocera* (White and Elson-Harris 1992). It is now clear that this distinction was falsely based on too limited taxon sampling within *Ceratitis*. Further studies are needed to determine reliable characters to separate these genera.

## Supplementary Material

XML Treatment for Ceratitis anonae

XML Treatment for Ceratitis fasciventris

XML Treatment for Ceratitis capitata
